# Fibrillin assemblies: extracellular determinants of tissue formation and fibrosis

**DOI:** 10.1186/1755-1536-3-24

**Published:** 2010-12-02

**Authors:** Jacopo Olivieri, Silvia Smaldone, Francesco Ramirez

**Affiliations:** 1 Scienze Mediche e Chirurgiche, Sezione Clinica Medica, Universita' Politecnica delle Marche, Ancona, Italy; 2 Pharmacology and Systems Therapeutics, Mount Sinai School of Medicine, New York, USA

## Abstract

The extracellular matrix (ECM) plays a key role in tissue formation, homeostasis and repair, mutations in ECM components have catastrophic consequences for organ function and therefore, for the fitness and survival of the organism. Collagen, fibrillin and elastin polymers represent the architectural scaffolds that impart specific mechanic properties to tissues and organs. Fibrillin assemblies (microfibrils) have the additional function of distributing, concentrating and modulating local transforming growth factor (TGF)-β and bone morphogenetic protein (BMP) signals that regulate a plethora of cellular activities, including ECM formation and remodeling. Fibrillins also contain binding sites for integrin receptors, which induce adaptive responses to changes in the extracellular microenvironment by reorganizing the cytoskeleton, controlling gene expression, and releasing and activating matrix-bound latent TGF-β complexes. Genetic evidence has indicated that fibrillin-1 and fibrillin-2 contribute differently to the organization and structural properties of non-collagenous architectural scaffolds, which in turn translate into discrete regulatory outcomes of locally released TGF-β and BMP signals. Additionally, the study of congenital dysfunctions of fibrillin-1 has yielded insights into the pathogenesis of acquired connective tissue disorders of the connective tissue, such as scleroderma. On the one hand, mutations that affect the structure or expression of fibrillin-1 perturb microfibril biogenesis, stimulate improper latent TGF-β activation, and give rise to the pleiotropic manifestations in Marfan syndrome (MFS). On the other hand, mutations located around the integrin-binding site of fibrillin-1 perturb cell matrix interactions, architectural matrix assembly and extracellular distribution of latent TGF-β complexes, and lead to the highly restricted fibrotic phenotype of Stiff Skin syndrome. Understanding the molecular similarities and differences between congenital and acquired forms of skin fibrosis may therefore provide new therapeutic tools to mitigate or even prevent disease progression in scleroderma and perhaps other fibrotic conditions.

## Introduction

The extracellular matrix (ECM) is a highly heterogeneous amalgam of morphologically diverse architectural entities composed of collagenous or elastic polymers, adaptor proteins and hydrophilic proteoglycans. The architectural matrix organizes and imparts structural integrity to individual tissues, in addition to modulating cell behavior by interacting with cell surface receptors and soluble growth factors. Primary or secondary dysfunctions in components of the architectural matrix can therefore interfere with both tissue integrity and cell performance. Cases in point are the fibrillin assemblies (microfibrils and elastic fibers), which represent the non-collagenous scaffolds of the architectural matrix. The present review focuses on the biology and pathophysiology of fibrillin assemblies, with a particular emphasis on recent evidence connecting fibrillin-1 with the control of TGF-β signaling and tissue fibrosis.

## Fibrillin assemblies and interactions

Fibrillins 1 and 2 are ubiquitous glycoproteins that self-polymerize into filamentous microfibrils with an average diameter of 10 nm in which individual molecules are organized in longitudinal head-to-tail arrays and associate laterally as well [1-4]. Fibrillin microfibrils can additionally serve as the structural template for tropoelastin deposition and/or crosslinking during elastic fiber formation. Specific segments of the fibrillins interact *in vitro *with numerous extracellular signaling and cell surface molecules, including fibronectin, fibulins, latent TGF-β-binding proteins (LTBPs), bone morphogenetic protein (BMP) pro-peptides, syndecans and integrins. The multiple molecular interactions of fibrillins are believed to drive the assembly of morphologically distinct macroaggregates, which contribute to imparting the structural integrity to individual tissues and organs (structural role), and to target TGF-β and BMP complexes to the architectural matrix, which contributes to instructing the behavior of cells (instructive role) TGF-βs and BMPs are potent modulators of ECM metabolism, that are under the control of a complex network of relays and servomechanisms operating within and outside the cell, and at the cell surface [5-7]. Extracellular control of local TGF-β and BMP signals - and -in particular the one that involves fibrillin microfibrils [3] - has recently emerged as a critical aspect of tissue formation, homeostasis and repair [6]. There is however, significant variability in how fibrillins can bind TGF-β and BMP complexes, and how fibrillin-bound TGF-β and BMP complexes can signal to cells.

TGF-β 1, 2 and 3 (hereafter collectively referred to as TGF-β) are secreted either as a small latent complex (SLC) in which bioactive homodimers are non-covalently associated with processed pro-peptides (latency-associated protein; LAP) or as a large latent complex (LLC) in which the TGF-β-SLC complex is bound to LTBPs [8]. Association with LAP blocks the ability of bioactive TGF-β dimers to interact with the cognate receptors TGFBR1 and TGFBR2, whereas binding to LTBPs directs TGF-β-SLC sequestration in the ECM through LTBP-mediated association with fibronectin fibrils first, and fibrillin assemblies subsequently [9,10]. Matrix metalloproteinases (MMPs), BMP1, thrombospondin-1, small proteoglycans and integrin receptors are involved in releasing latent TGF-β from the ECM by modifying LLC structure or disrupting LAP-mediated latency [11]. Thus, latent TGF-β complexes bind indirectly to extracellular microfibrils and as a result, fibrillin-bound TGF-β-LLC requires a two-step activation process to signal; i.e.: release from the ECM and LAP dissociation.

BMPs are also secreted and targeted to the ECM as crosslinked dimers non-covalently associated with the pro-peptides that can interact in vitro with the N-termini of fibrillin-1 and -2 [12,13]. In contrast to TGF-β, however, BMP pro-peptides do not generally confer latency to the associated dimers, and as a result, BMPs can readily signal once released from the ECM [14]. Accordingly, the fibrillins act as storage scaffolds that distribute, concentrate and confer latency to BMPs, while soluble antagonists and their modulators are probably the only extracellular molecules that control the activity matrix-unbound (free) BMPs. Hence, BMP complexes bind directly to extracellular microfibrils, and signaling by fibrillin-bound BMPs solely involves the step of releasing the ligand from the matrix.

Fibrillins 1 and 2 share an Arg-Gly-Asp (RGD) sequence within a flexible loop in the middle of the molecules, which favors binding to integrins α_5_β_1 _and α_v_β_3/6 _[15-18]; additionally, the N-terminal third of fibrillin-2 contains a second RGD sequence [2]. An *in vitro *model of platelet-derived growth factor-induced fibroblast migration suggests that the common RGD sequence of fibrillins induces more lamellipodia and more widespread remodeling of the leading edge, whereas the unique RGD motif of fibrillin-2 stimulates migration with greater directional persistence [19]. Additionally, evidence indicates that a high affinity heparin-binding site located immediately next to the RGD site of fibrillin-1 enhances integrin-mediated cell adhesion, probably by binding syndecan receptors [20]. There is reason to believe that the interactions between fibrillins and cell receptors and between fibrillin and fibronectin fibrils occur in the pericellular space, where they probably guide both the early steps of fibrillin polymerization and the targeting of growth factors to extracellular microfibrils [9,10,21-26]. Integrins α_v_β_5/6/8 _can also participate in latent TGF-β release from the matrix and/or activation through proteolytic and non-proteolytic mechanisms [27-29]. Thus, pericellular release of ECM remodeling signals appears to be locally integrated with microfibril biogenesis and growth factor targeting to the matrix.

## Fibrillin diseases in humans

Heterozygous mutations in fibrillin-1 or fibrillin-2 cause two clinically related disorders of the connective tissue, MFS (OMIM 154700) and congenital contractural arachnodactyly (CCA: OMIM 121050) respectively [30,31]. MFS is predominantly characterized by abnormalities in the ocular, skeletal and cardiovascular systems. Deficiencies in the cardiovascular system, particularly development of aortic root dilatation, are the main cause of mortality and morbidity in affected patients [30]. The main clinical features of CCA include joint contractures, crumpled ears and musculoskeletal manifestations; cardiovascular and gastrointestinal anomalies are occasionally present in infants with a severe/lethal form of the disease [31]. On the one hand, the multiple traits of MFS and CCA underscore the importance of fibrillin-1 and fibrillin-2 microfibrils in the formation and function of several organ systems. On the other hand, the distinct phenotypes of MFS and CCA imply that fibrillin-1 and -2 have discrete functions in spite of participating in the same architectural assemblies. The functional diversity of fibrillins -1 and -2 involves both their structural contribution to microfibril integrity, as the two proteins display temporally distinct expression patterns, and their instructive contribution to cell performance, as loss of function mutant mice show discrete tissue-specific TGF-β and/or BMP-dependent abnormalities (see below) [32].

Mutations in the human fibrillin-1 gene (*FBN1*) are particularly interesting because they are associated with a wide spectrum of clinical severity in MFS, irrespective of where they are located in the protein or how much they reduce gene expression [30]. This apparent lack of genotype-phenotype correlations also applies to the occasional *FBN1 *mutations in patients who do not fulfill the strict diagnostic criteria of MFS [33]; these rare instances include familial ectopia lentis (OMIM 129600), Shprintzen-Goldberg syndrome (OMIM 182212) and Weill-Marchesani syndrome (OMIM 608328) [34-36]. By contrast, there is a very strong correlation between mutations affecting a specific domain of fibrillin-1 and the unique phenotype of stiff skin syndrome (SSS; OMIM 184900) [37]. SSS mutations cluster around the sole integrin-binding RGD sequence of fibrillin-1, and lead to a highly restricted (as opposed to a systemic) phenotype that resembles a congenital form of scleroderma. The domain-specific nature and unique phenotypic outcome of SSS mutations suggests a different pathogenetic mechanism from that of MFS. Clinical and pathogenetic relationships between SSS and scleroderma are discussed more extensively in the last section of this review.

## Fibrillin mutations in mice

Several mice with mutations in the *Fbn1 *or *Fbn2 *gene have been created that replicate the clinical features of MFS, SSS and CCA. Mutations in *Fbn1 *include those that blunt gene expression and lead in homozygosity to progressively severe (*Fbn1^mgR/mgR ^*mice) or neonatal lethal MFS (*Fbn1^-/- ^*mice) and those that perturb protein structure and lead in heterozygosity lead to mild (*Fbn1^C1039G/+ ^*mice) or subclinical MFS (*Fbn1^GT-8/+ ^*mice) [38-41]. Loss of fibrillin-2 synthesis in *Fbn2^-/- ^*mice is associated with several CCA traits, such as joint contractures, osteopenia and muscle weakness and atrophy, as well as a unique limb patterning defect not seen in either CCA patients or *Fbn1^-/- ^*mice, namely digit fusion (syndactyly) with involvement of either soft or hard tissue [42]. Lastly, a spontaneous tandem duplication within *Fbn1 *is responsible for the phenotype of tight skin (*Tsk/+*) mice, which includes myocardial, skeletal and pulmonary abnormalities of MFS and fibrotic features of SSS (see below) [43].

Characterization of mice harboring mutations in *Fbn1 *and/or *Fbn2 *has provided invaluable insights into microfibril biogenesis and function. The first insight indicated that fibrillins -1 and -2 perform partially overlapping structural functions in developing and mature tissue. This conclusion is based on the findings that *Fbn2 *expression is largely restricted to the forming and remodeling tissues, that fibrillin-2 proteins are buried within postnatal microfibrils, and that the vascular phenotype and average survival of *Fbn1^-/-^*;*Fbn2^-/- ^*or *Fbn1^-/-^*;*Fbn2^-/+ ^*mice are significantly more severe than those of *Fbn1^-/- ^*or *Fbn2^-/-^*mice, [40,42,44,45]. Taken together, these data imply that fibrillin-2 polymers form the inner scaffold that supports the deposition and/or organization of fibrillin-1 polymers, and that continued and proper deposition of fibrillin-1 is absolutely required for the postnatal maturation and mechanical compliance of aortic tissue.

**Figure 1:**
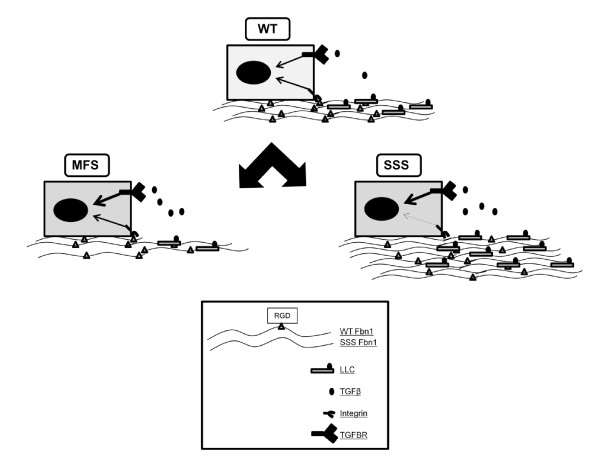
**Hypothetical models of MFS and SSS pathogenesis**. The top drawing depicts the normal wild-type (WT) condition in which a cell interacts with fibrillin-1 microfibrils through RGD-mediated integrin binding and through TGF-β signals released from the fibrillin-1 microfibrils (thin arrows). Mutations in MFS decrease microfibril deposition/stability and promote excessive TGF-β signaling (thick arrow). Mutations in stiff skin syndrome perturb integrin-mediated microfibril deposition and increase matrix-directed TGF-β signaling (thick arrows).

The second insight that has emerged from the studies of *Fbn *mutant mice is that the organization of fibrillin-1 and fibrillin-2 polymers within the forming and mature microfibrils also determines the contextual regulation of local TGF-β and BMP signals. Vascular and bone findings support this contention. Aortic aneurysm progression in *Fbn1^C1039G/+ ^*and *Fbn1^mgR/mgR ^*mice is largely driven by increased latent TGF-β activation and signaling secondary to loss of TGF-β-LLC sequestration in the ECM (Figure 1), a molecular phenotype that is replicated in cultured primary smooth muscle cell isolated from *Fbn1 *mutant aortas [46,47]. By contrast, improper TGF-β signaling is not seen in either the aortas or cultured vascular smooth muscle cells from *Fbn2^-/- ^*mice [40,47]. Hence, fibrillin-1, but not fibrillin-2, restricts TGF-β activity in the medial layer of the postnatal aorta by interacting with LTBP components of the LLC. Likewise, the unique presence of syndactyly in *Fbn2^-/- ^*mice and its apparent association with decreased BMP signaling are strong indications that fibrillin-2, but not fibrillin-1, promotes BMP activity in the developing autopods [42]. Bone remodeling is yet another example of a tissue-specific program in which fibrillin -1 and -2 contribute differently to the extracellular regulation of local TGF-β and BMP signals [48-50]. Both *Fbn1^-/- ^*and *Fbn2^-/- ^*osteoblast cultures display enhanced TGF-β signaling, which inhibits osteoblast maturation, but only cultured osteoblasts from *Fbn1^-/- ^*and *Fbn1^mgR/mgR ^*mice exhibit higher than normal BMP activity, which promotes osteoblast maturation [48,49]. As a result, osteoblasts deficient for fibrillin-2 fail to mature properly, and bone formation is impaired in *Fbn2^-/- ^*mice, whereas osteoblast maturation and bone formation are largely unaffected in *Fbn1^-/- ^*and *Fbn1^mgR/mgR ^*mice [48,49]. Increased TGF-β signaling, however, upregulates Rankl production by osteoblasts, with consequent stimulation of bone resorption by osteoclasts in mice deficient in either fibrillin-1 or fibrillin-2 deficient mice [49,50]. Collectively, these genetic findings indicate that microfibrils regulate local TGF-β and BMP signals in a manner that is stage-, tissue-, ligand- and fibrillin-specific. The mechanistic basis for this specificity contextual remains to be determined.

## Fibrillin-1 and scleroderma

Tissue fibrosis is traditionally viewed as the pathological counterpart of physiological wound healing [51]. In response to insult(s) altering ECM integrity or architecture, activated mesenchymal cells initiate a matrix-remodeling program in which anabolic and catabolic activities are tightly balanced. In fibrotic conditions, such as scleroderma, cell insensitivity to normal regulatory signals leads to excessive ECM deposition and ultimately, organ failure [51,52]. Past efforts have mostly focused on characterizing the molecular mechanisms responsible for aberrant cell behavior and less attention has been paid to defining the extracellular determinants of tissue fibrosis. This situation has rapidly changed because new evidence from acquired and congenital forms of human scleroderma and *Tsk/+ *mice has implicated dysfunction of fibrillin-1 microfibrils in skin fibrosis [53].

Widespread tissue fibrosis is the hallmark of systemic sclerosis (SSc), the most common form of acquired scleroderma [54,55]. Thickening and hardening of the skin is progressive, and evolves from the extremities to the trunk in a centripetal manner that rarely affects the back. In contrast to other fibrotic disorders, autoimmunity and vasculopathy characteristically precede SSc fibrosis [56,57]. Skin fibrosis begins near to blood vessels in the reticular dermis, and is accompanied by a prominent inflammatory infiltrate [58-60]. Fibroblasts explanted from involved sites show a transiently activated phenotype characterized by elevated expression of ECM components, MMP inhibitors (TIMPs) and adhesion molecules, in addition to constitutive TGF-β signaling [51]. Consistent with this last finding, SSc biopsies display increased activation of the Smad2/3 pathway, and skin fibrosis is observed in transgenic mice over-expressing constitutively active TGFBR1 [61,62]. There is also evidence suggesting that perivascular monocellular infiltrate may be responsible for exaggerated TGF-β activity, and that activated SSc fibroblasts may themselves augment TGF-β signaling by increasing TGFBR expression [63,64]. Alteration of LLC storage in the ECM may also promote the increase in TGF-β signaling as well. Indeed, SSc fibroblasts secrete and assemble microfibrils that appear to be unstable, and SSc biopsies exhibit disorganized fibrillin-1 aggregates and fragmented elastic fibers throughout the dermis [65-67]. Moreover, *FBN1 *polymorphisms characterize high SSc incidence in Choctaw Indians and a smaller cohort of Japanese patients [68,69]. Lastly, circulating autoantibodies against fibrillin-1 have been described in SSc patients, that can induce the molecular signature of activated cells in healthy fibroblasts, apparently through a TGF-β-dependent mechanism [70].

As already mentioned, *Tsk/+ *mice replicate some MFS features (bone lengthening, cardiac hypertrophy and lung emphysema) as well as characteristic SSc features (skin fibrosis with excessive accumulation of collagen I and microfibrils, and the emergence of circulating autoantibodies against topoisomerase I, RNA polymerase I and fibrillin-1) [71-74]. Additional pathological correlates between the human and mouse phenotypes include the ability of TGF-β interference to reduce skin thickness, either genetically by pairing the *Tsk *allele with *Tgfβ *haploinsufficiency or pharmacological inhibition of Smad3-mediated transcription in *Tsk/+ *mice [75,76]. Furthermore, expression of the *Tsk *protein in mouse embryonic fibroblasts is accompanied by excessive collagen I deposition along with up-regulation of the microfibril-associated protein MAPG2, which is over-expressed in SSc skin [77,78]. There are also key differences between the two phenotypes. First, *Tsk+ *mice lack the microvascular involvement characteristic of SSc [79]; second, fibrosis is still apparent in immunodeficient *Tsk/+ *mice [80]; and third, the circulating autoantibodies in *Tsk/+ *mice emerge relatively late compared with the onset of skin fibrosis [71]. Moreover, the fibrotic involvement in *Tsk/+ *mice follows a different pattern from that in SSc, as skin thickening begins after 2-4 weeks, progresses slowly and is more evident in the interscapular area and is absent from regions devoid of fascia, such as the ear pinna [81,82]. Lastly, histological analyses have confirmed the relative normalcy of dermal thickness and collagen content in *Tsk/+ *skin, in addition to attributing the fibrotic phenotype to a marked hyperplasia of the loose subdermal connective tissue, which results in increased tethering of the skin to the underlying muscle layer [81].

The discovery of *FBN1 *mutations in SSS has provided a more cogent argument for the causal relationship between altered microfibril biogenesis, increased TGF-β signaling, and skin fibrosis [37]. SSS is an extremely rare congenital disease (~40 cases reported to date) that manifest in infancy or in early childhood with rock-hard skin bound firmly to the underlying tissues [83,84]. Skin manifestations are most prominently observed in areas with abundant fascia, such as the buttocks, thighs, and shoulder girdle area. However, there is some disagreement about the specific area affected, sometimes being reported as the dermis and in a few cases as the underlying fascia [37,85-87]. It should also be noted that histological studies have rarely assessed hypodermal tissue because this examination requires more invasive procedures [85]. Early clinical presentation of SSS often impacts skeletal growth and results in deformities such as scoliosis, a tiptoe gait, and a narrow thorax that can ultimately impair pulmonary function and lead to respiratory distress [84,88]. In most cases, the disease progresses slowly and is not fatal. Lack of visceral involvement, immunologic abnormalities and vascular disturbances differentiate this inherited condition from rare cases of pediatric SSc [84,89].

In spite of the above considerations, there is reason to believe that elucidating the molecular underpinning of skin fibrosis in SSS patients and *Tsk/+ *mice could yield general principles of disease progression in SSc. SSS mutations cluster around the sole integrin (RGD)-binding site of fibrillin-1, whereas the *Tsk *mutation generates longer fibrillin-1 molecules with duplicated RGD sequences [37,43]. Current evidence indicates that altered cell-matrix interactions in SSS have multiple negative effects on ECM assembly, TGF-β activity, cell identity and integrin signaling [37]. Specifically, perturbed integrin-directed fibrillin-1 assembly in SSS leads to excessive microfibril deposition and consequently, greater latent TGF-β concentration and signaling (Figure 1). Increased TGF-β activity in turn induces keratinocytes to lose their phenotype and become activated mesenchymal cells (epithelial to mesenchymal transition; EMT), an observation in line with keratinocyte contribution to hypertrophic scar formation [90]. Additionally, loss of RGD binding also perturbs microfibril-induced integrin signaling, that normally directs several cellular activities during skin development (Figure 1). The larger size of fibrillin-1 together with duplicated RGD sequences may trigger the same cascade of events in the skin of *Tsk/+ *mice, in addition to eventuating MFS-like manifestations in other organ systems by mechanism(s) that influence other aspects of fibrillin-1 assembly and function [43,91,92]. In line with this argument, the pathogenesis of reduced bone mass in *Tsk/+ *mice differs from that of *Fbn1^mgR/mgR ^*mice, as impaired bone formation and increased bone resorption in the latter [49,93]. The difference are likely to be accounted for by unopposed elevation of TGF-β signaling in *Tsk/+ *bones and by balanced augmentation of both TGF-β and BMP activity in the *Fbn1^mgR/mgR ^*counterparts [49]. Further analyses of *Tsk/+ *mice and creation of SSS mice promises to elucidate the pathogenetic contribution of fibrillin-1 microfibrils to fibrotic phenotypes.

## Conclusions and perspectives

Fibrillin assemblies represent a nodal point that integrates the biological network of structural and instructive information flowing to and from the cell, which orchestrates tissue formation, homeostasis and repair. Such an integrated view of the molecular interactions within fibrillin assemblies and between them and the resident cells has expanded our understanding of the roles of the architectural matrix substantially, in addition to providing new means to test evidence-based therapies in conditions characterized by primary or secondary defects in fibrillin assemblies. In this light, TGF-β immediately emerged as the first network component to be targeted by pharmacological interventions aimed at improving aortic aneurysm progression in MFS. The strategy was based on the prior knowledge that blockade of angiotensin II receptor I (AT1R) activity reduces excessive TGF-β signaling in experimental renal and cardiac fibrosis [94,95]. within accordance with this prediction, losartan treatment was shown to restore aortic wall architecture in *Fbn1^C1039G/+ ^*mice and to mitigate aortic root dilation in a small cohort of children with severe MFS [46,96]. A more recent study has confirmed the efficacy of losartan treatment in improving aortic wall degeneration in *Fbn1^mgR/mgR ^*mice, a more severe model of MFS than the *Fbn1^C1039G/+ ^*mouse, even though the regimen showed no beneficial impact to counteract bone loss [49]. These findings, together with evidence suggesting that some individuals may not respond to losartan as effectively as others [97,98], strongly argue for a multifaceted treatment strategy in MFS and perhaps, in related disorders of the connective tissue. Indeed, *in vivo *data have demonstrated that broad inhibition of tyrosinekinases is a more efficient strategy to control tissue fibrosis than using specific receptor inhibitors [99-101]. Likewise, *in vivo *and *ex vivo *lines of evidence have implicated additional contributors to vascular disease onset and/or progression in MFS. First, studies of *Fbn1^-/- ^*aortas and vascular SMC suggest that stress responses triggered by a structurally deficient matrix and mediated by ROS through the Ras/mitogen-acivated protein kinase signaling pathway participate in Smad2/3 activation independently of TGF-β [47]. Second, improved aortic wall architecture in *Fbn1 *mutant mice systemically treated with doxycycline implies that improper MMP activity (and conceivably TIMP activity as well) exacerbates TGFβ-driven aneurysm progression in MFS [102,103]. Third, paradoxical increase of TGFβ signaling in the Loeys-Dietz syndrome (LDS; OMIM-609192), which is caused by heterozygous loss-of-function mutations in TGFBR1 or TGFBR2 [104], points to the potential impairment of TGFβ auto-regulation, compensatory mechanisms and/or alternative signaling cascades [30,32]. Lastly, domain-specific mutations in SSS correlate perturbations in ECM assembly and cell-matrix communication with triggering a wide array of signals that stimulate and sustain fibrosis [37]. TGF-β and EMT antagonism on the one hand, and integrin agonism on the other, have therefore emerged as therapeutic strategies to be tested alone or in mutant mice that model human SSS [37].

Moreover, the recent report that lymphatic and blood endothelial cells participate in fibrillin-1 deposition in human skin raises the intriguing possibility of a microvascular origin of dermal fibrosis in SSS [105]. It is therefore safe to conclude that the study of SSS patients and mice will benefit our understanding of SSc pathogenesis, despite the noted differences in clinical presentation and natural history between these two diseases.

## List of abbreviations

CCA: congenital contractural arachnodactyly; ECM: extracellular matrix; EMT: epithelial-to-mesenchymal transition; *FBN *and *Fbn*: human and mouse fibrillin genes, respectively; LAP: latency associated protein; LDS: Loeys-Dietz syndrome; LLC: large latent complex; LTBP: latent TGFβ-associated protein; MFS: Marfan syndrome; MMP: matrix metalloproteinase; SSc: Systemic Sclerosis; SSS: Stiff Skin syndrome; TGFBR: TGFβ receptor; *Tsk*: tight skin mutation.

## Competing interests

The authors declare that they have no competing interests.

## Authors' contributions

JO, SS and FR drafted the manuscript. FR was responsible for its design and coordination. All authors read and approved the final product.
